# Tracking 20 Years of Compound-to-Target Output from Literature and Patents

**DOI:** 10.1371/journal.pone.0077142

**Published:** 2013-10-29

**Authors:** Christopher Southan, Peter Varkonyi, Kiran Boppana, Sarma A.R.P. Jagarlapudi, Sorel Muresan

**Affiliations:** 1 TW2Informatics, Göteborg, Sweden; 2 Discovery Sciences, Chemistry Innovation Centre, AstraZeneca R&D Mölndal, Mölndal, Sweden; 3 GVK Biosciences Pvt. Ltd., Hyderabad, India; 4 Food Control Department, Banat's University of Agricultural Sciences and Veterinary Medicine, Timisoara, Romania; University of South Florida College of Medicine, United States of America

## Abstract

The statistics of drug development output and declining yield of approved medicines has been the subject of many recent reviews. However, assessing research productivity that feeds development is more difficult. Here we utilise an extensive database of structure-activity relationships extracted from papers and patents. We have used this database to analyse published compounds cumulatively linked to nearly 4000 protein target identifiers from multiple species over the last 20 years. The compound output increases up to 2005 followed by a decline that parallels a fall in pharmaceutical patenting. Counts of protein targets have plateaued but not fallen. We extended these results by exploring compounds and targets for one large pharmaceutical company. In addition, we examined collective time course data for six individual protease targets, including average molecular weight of the compounds. We also tracked the PubMed profile of these targets to detect signals related to changes in compound output. Our results show that research compound output had decreased 35% by 2012. The major causative factor is likely to be a contraction in the global research base due to mergers and acquisitions across the pharmaceutical industry. However, this does not rule out an increasing stringency of compound quality filtration and/or patenting cost control. The number of proteins mapped to compounds on a yearly basis shows less decline, indicating the cumulative published target capacity of global research is being sustained in the region of 300 proteins for large companies. The tracking of six individual targets shows uniquely detailed patterns not discernible from cumulative snapshots. These are interpretable in terms of events related to validation and de-risking of targets that produce detectable follow-on surges in patenting. Further analysis of the type we present here can provide unique insights into the process of drug discovery based on the data it actually generates.

## Introduction

The declining productivity of global pharmaceutical development, measured in terms of small-molecule New Chemical Entities (NCEs) factored against their rising cost, has been extensively analysed and commented on in recent years [1], [2], [3],[4],[5]. Although the longer term approval rate has been projected as closer to linear than a real decline [1], the attrition rates from 1990 to 2004 have continuously increased for every development phase [3]. Assessing information and statistics about the progression of NCEs is possible because by the time candidates enter clinical trials they have usually been declared in publications, meeting reports, news releases and portfolio listings. As they approach regulatory submission they will also have been assigned an International Non-Proprietary Name (INN) [6] and national equivalents thereof, such as the USAN. The statistics quoted by competitive intelligence sources that collate such data vary in exact numbers, but suggest approximately 35,000 compounds have ever entered development and the 2010 figure of 9,737, was nearly 10-fold higher than for 1980 [7]. A more open and specific count of historically advanced drug candidates can be obtained by performing a query in PubChem Compound for INN or USAN. This retrieves 10421 structures (June 2013). Thus, for R&D, while there is adequate data to measure the “D” output an equivalent assessment of “R” (as the input to “D”) is much more difficult. The main reason is because the data generated by commercial organisations that dominate global output is considered proprietary, even though the continuing imperative for this has been challenged [8].

Notwithstanding this information shadow that extends back into the research phase, patents and journal publications provide valuable surrogate outputs. We have been unable to find any formal description of the information flow between these two document types but it can be briefly described as follows. Drug discovery project teams typically apply for patents to claim and protect the chemical space around their lead series from which clinical development candidates may be chosen [9]. This sets the minimum time between the generation of data and its disclosure to 18 months. In practice, this is usually extended, not only by the time necessary for collating the data and drafting the application but also where strategic choices may be made to file later in the development cycle to maximise the patent term. It is also common to file separate applications for each distinct chemical series the team is progressing.

While some drug discovery operations may eschew non-patent disclosure entirely, it is nevertheless common practice (and has business advantages) for project teams to submit papers to journals that include some of the same structures and data from their patents. While the criteria for inventorship are different than for authorship, there are typically team members in-common between the two types of attribution. Journal publications may or may not identify the lead compound by linking the structure to a code name, depending on how far this may have progressed as a clinical candidate [10].

The time lag can vary between submitting manuscripts immediately after filing, waiting until the application has published, deferring publication until a project has been discontinued, or the code name may never be publically resolvable to a structure. A recent comparison showed that 6% of compound structures exemplified in patents were also published in journal articles [11]. While the patterns described above will be typical for pharmaceutical and biotechnology companies, the situation in the academic sector differs in a number of respects [12]. Universities and research institutions are publishing increasing numbers of patents for bioactive compounds but their embargo times for publication and/or upload of screening results to open repositories, such as PubChem BioAssay, are generally shorter [13].

So what is the global research phase output of compounds across time directed against protein targets for human disease? There is no current public information source that can supply a definitive answer because this is associated with complex data mining challenges, particularly for the patent component. However, data related to the wider question is becoming more accessible. For example, the query interfaces at major patent office portals have improved considerably over the last few years (EPO Espacenet [14], USPTO [15], and WIPO [16]). In addition, there has been a proliferation of open resources for full-text patents such as Free Patents Online [17] and Google Patents [18]. These have recently been joined by new public resources that include chemical structures extracted from patents. These include extractions from US patents in SCRIPDB [19], the deposition from IBM of structures from pre-2000 patents into PubChem [20], selected European patent structures added to PubChem by the SLING Consortium [21], and the recent release of SureChemOpen [22].

Notwithstanding the utility of these resources, they cannot be used to extract the explicit mapping between compounds and targets with high specificity at large-scale. The obstacles to this include identifying representative patents, unambiguous resolution of target names, and establishing which example structures are linked to what activity data. Last but not least, these challenges are compounded by the common strategic practice for patents to be written in a style that obfuscates data relationships, even if these may later be reformulated in journal papers with the necessary clarity to pass peer-review. In regard to the extraction of structures, activity data and protein targets from papers, the largest public source is ChEMBL to which links are available from PubChem BioAssay, European PubMed Central and other databases [23], [24], [25].

In addition to public sources there are a number of companies whose business models are based on brokering drug research and/or development data extracted from patents, papers and other public sources. Many of these declare their basic entity counts and, by definition, their products provide functionality for subscribers to query the content. However, research output statistics at the magnitude and detail we are considering here continue to be difficult to retrieve with high precision from any single source, public or commercial. This is because of both insufficient integration of data relationships and the extractions are not carried out at a large enough scale [26].

As a prelude to comparing the two, it is useful to note the different time scales for “R” *vs.* “D” quoted in the literature. Significantly, the averages have moved up, from 9.7 years during the 1990's, to 13.9 years for products launched from 2000 onwards [3]. The research phases have been estimated at approximately 5.5 years from hit-to-candidate with a subsequent average clinical development phase of 6 years [2]. The timing of chemical patenting within this research span varies considerably depending on organisational or project-specific choices. For example, the decision may be made for an early defensive filings with a lead series. Alternatively this may be delayed until well after optimisation to maximise patent term [27]. Regardless of these uncertainties, we can assume that NCE drugs approved during 2012 will, on average, have had their first composition-of-matter patents published approximately 10 years previously.

In this work we have undertaken an assessment of early-phase drug discovery output. We have been able to achieve this using data compiled from structure-activity-target relationships extracted from over 140,000 patents and papers extending over more than 40 years. In a previous report, we used this data corpus to rank 1654 protein targets by the number of compounds directed against them [29]. The difference in this study is that we now analysed the same corpus (updated by two years) but, rather than the cumulative end-point statistics, we focus here on following entity changes on a year-on-year basis. This is possible because the data structure enables every protein identifier and compound to be linked to the publication year of each document it was extracted from. Earlier versions of similar data sets to those used in this work (i.e. also using the GVKBIO databases) have been dissected according to institution, target numbers, target classes and molecular properties against time [27], a single journal across time [28], and, from a cumulative snapshot, the ranking of all targets by compound numbers *vs.* molecular scaffolds [29].

We have restricted out tracking of research output to the 20 years of 1991 to 2010 for three reasons. Firstly, it encompasses the distinct global growth phase. Secondly, we can rely on the completeness of data collection up to 2010 (i.e. without any significant back-log from source feeds or curatorial triage). Thirdly, this period covers the completion of the human genome along with major advances in screening technology. Lastly, it covers the development time for recently approved drugs as well as the early research efforts directed against their targets. Our approach starts with the discernment of major time patterns for compounds and targets. By selecting corroborative data sources and ancillary analyses we test some possible explanations for these patterns. We then focus on an individual institution and, lastly, track six individual targets on the same year-by-year basis.

## Results

### Cumulative statistics

The use of the word “compound” for a generally lead-like chemical structure needs no qualification here, except to point out that the data set encompasses a significant proportion beyond the typical small-molecule range of MWs above 1000. These are typically peptidic protease inhibitors, antibiotics and complex natural products. We have previously discussed the imprecision and equivocality associated with the term “target” in some detail [29]. In summary, the comprehensive capture of compound-to-protein relationships encompasses a significant proportion that are not considered as drug targets *per se*, for example those derived from paralogue and orthologue cross-screening. It is thus more correct to classify the data as compound-to-protein mappings. However, for convenience, we continue to use “target” as the umbrella term for the protein identifiers.

Our first task was to update the statistics for the data sets we used for our 2011 analysis [29]. The current figures in Table 1 provide the new cumulative totals from 1945 for journals and 1966 for patents up to November 2012.

**Table 1:** Content statistics of MCD and TCD, populated from journal papers and patents, respectively.

Entity Type	Count
**1.** Journal articles	82,146
**2.** Patents	58,809
**3.** Compounds from journals	1,007,340
**4.** Average number of compounds per journal article	12.3
**5.** Compounds from patents	2,702,397
**6.** Average number of compounds per patent	46
**7.** Compounds from journals and patents	3,566,264
**8.** Human targets with protein IDs from journals	1,759
**9.** Human targets with protein IDs from patents	1,401
**10.** Human targets with protein IDs from journals and patents	1,987
**11.** All targets with protein IDs from journals	4,084
**12.** All targets with protein IDs from patents	2,676
**13.** All targets with protein IDs from journals and patents	4,628

Compound counts are unique chemical structures for the specified source or source combination. All protein identifiers are linked to quantitative assay results which include values for IC50, pIC50, Ki or % inhibition. This data was retrieved from the GOSTAR database that integrates MCD and TCD with other GVKBIO database products [55].

**Figure 1:**
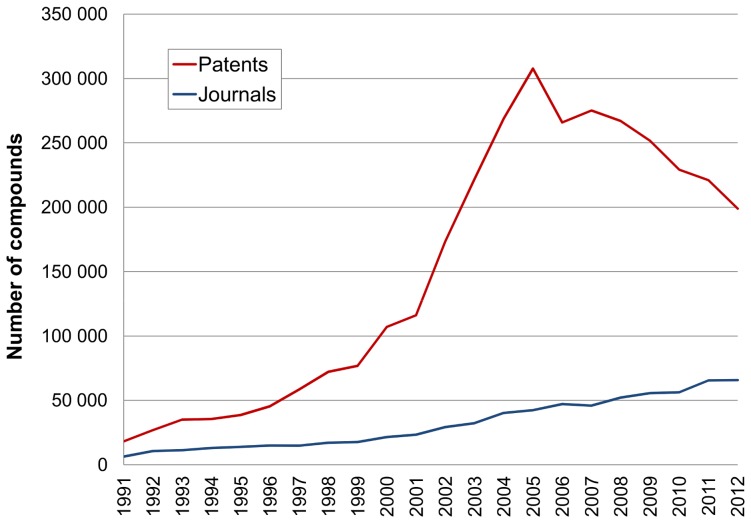
Compounds linked to human protein targets. These were extracted by year from patents (red) and journals (blue).

The first analysis results of plotting SAR-linked compound output from patents and papers are show in Figure 1. For patent chemistry output three distinct phases can be discerned. The first is a steady increase up to the 1990s. The second is acceleration between 2001 and 2005. However, this is followed by a fall from 2005 to 2012 amounting to ∼35%. In comparison, journal outputs show a steady increase over the entire period, although there are suggestions of an acceleration between 2001 and 2007, together with a subsequent slowdown. Additional plots were generated to test if the stringency of target mapping made any difference (results not shown). Regardless of whether compound plots were generated by the inclusion of all target species, human, mouse and rat or human only, the curve shapes were essentially the same.

**Figure 2:**
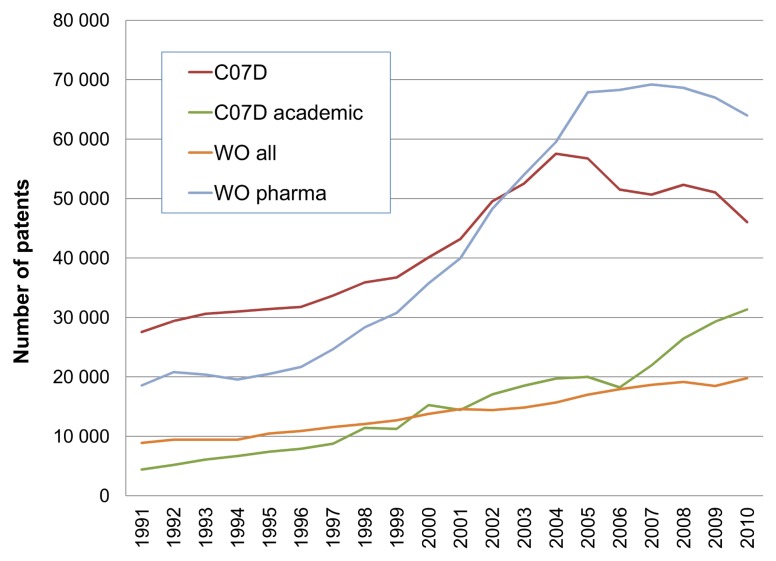
Patent office document counts. The light blue and orange plots are data from WIPO IP Statistics Data Centre [50]. These were selected as total world-wide patent applications for all categories (orange WO all, but divided by 10 for scaling) or the technology category pharmaceuticals (blue WO pharma). The other two plots were generated using the Espacenet advanced search interface [51]. With the selection of the IPC code C07D the red line is the total and the green line added a selection for “university OR institute” in the applicant field. For 2010 the numbers retrieved were 47,884 for C07D, 1,189 of these with “institute” and 2,366 with “university”.

While the inference is a decline of patented chemistry output related to drug discovery research we sought corroborative data to support this. We thus compared, over the same time scale, selected document counts for metadata queries from major patent office portals. These are the primary sources that feed into the GVKBIO triage for data extraction but are independent from it. We plotted four counts shown in Figure 2.

**Figure 3:**
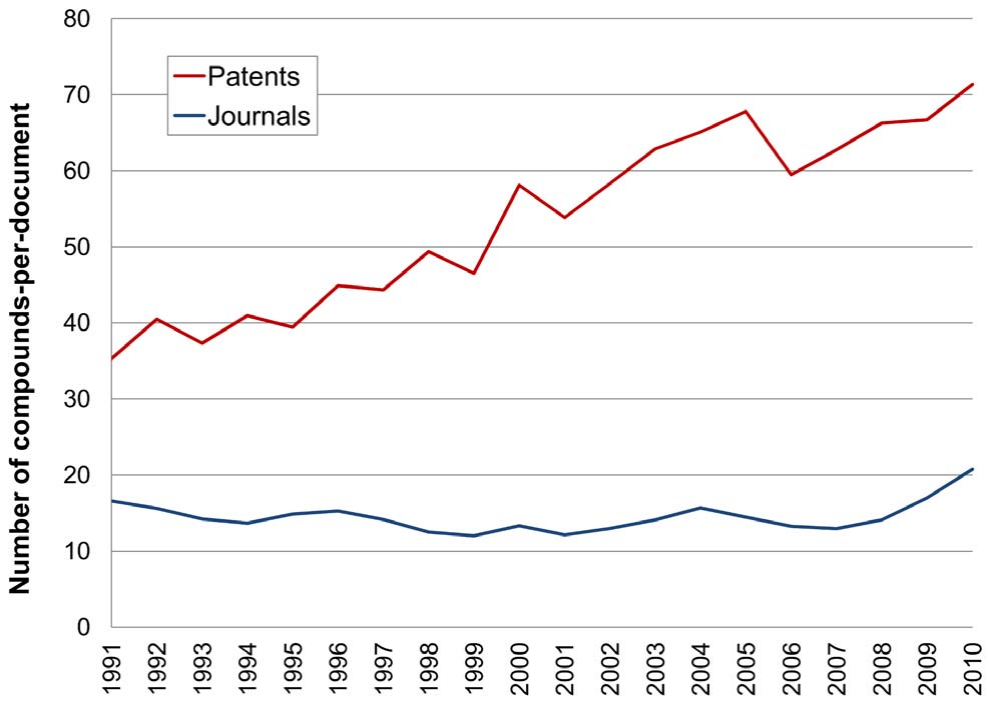
Average compounds-per-document by year from patents (red) and journals (blue).

We can use the patent office primary data in Figure 2 for hypothesis-testing the derived extraction data in Figure 1. Firstly, the post-2005 drop in compound output approximately parallels the selections for medicinal chemistry and pharmaceutical patents. The correlation thus infers that the downturn in Figure 2 is specific for (or at least dominated by) pharmaceutical research output. The rise in academic C07D patents, the International Patent Classification (IPC) code under which medicinal chemistry patents are usually filed, suggests the absence of confounding trends (i.e. no overall decline of non-pharma medicinal chemistry filings). As an obvious control data set, global patent output shows a constant increase over the same 20 years. Further inference testing was done by examining correlation between compounds and documents. As expected, document counts closely paralleled the compound profiles (results not shown) but it was also necessary to check for possible confounding changes in compounds-per-document. These results are plotted in Figure 3.

As the cumulative average since 1976 is 45 (Table 1) we can discern an approximate doubling of compounds-per-patent over the 20 years (Figure 3). In the context of declining output, this seems a counter-intuitive result. However, on the basis of a per-document average alone, we cannot discriminate between alternative interpretations of either, more examples *per se* being included or, an increase in the proportion of linked SAR data. However, our familiarity with the curation triage leads us to suggest the latter. Because the same source was used, the patent figures are comparable to those published for a molecular property study that included assignee comparisons [27]. In particular for that study, the calculated per-patent averages for WO filings covering the period 2001 to 2007 were AstraZeneca 57, GlaxoSmithKline 58, Merck 60, and Pfizer 71. These are broadly in line with the moving averages in Figure 3 but add the observation that individual assignee differences can be significant.

The compounds-per-paper numbers contrast with the increase for patents. While this averages out for papers at 12 (since 1945 in Table 1) it has risen to above 20 in 2010. Notably these numbers can be compared with other journal curation efforts. The latest releases of the World of Molecular Bioactivity WOMBAT database [30] indicates 18 compounds per-paper with ChEMBL (release 16) coming in at 17 compounds per-paper [31]. Considering the likelihood of differences in curation triage, SAR selection and journal choice in these independent operations (GVKBIO, WOMBAT and ChEMBL), this indicates both some consistency for expert extraction and a degree of long-term uniformity, at least in terms of SAR density in the medicinal chemistry literature. This consistency becomes important where journal outputs show small but interpretable changes for individual targets.

### Total Targets

**Figure 4:**
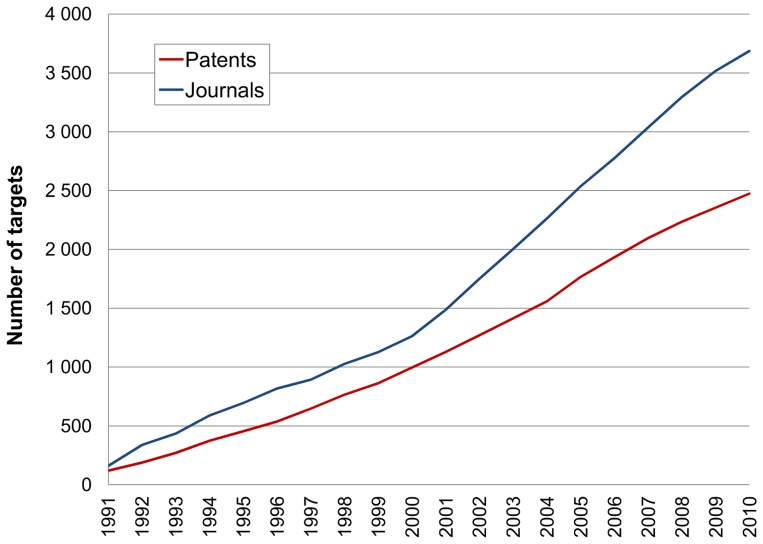
Cumulative targets. These are the additive totals up to each year, for proteins specified in patents (red) and journals (blue) from all species.

**Figure 5:**
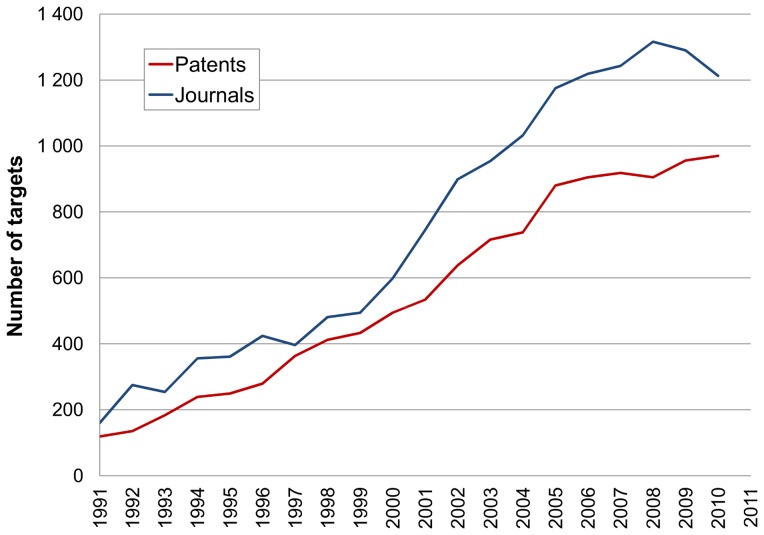
Year-on-year target counts. Each total represents the target proteins specified in the patents (red) and journals (blue) published in that year.

Our first assessment was to follow all protein identifiers across the 20 years. Results are shown for the cumulative totals (Figure 4) and the incremental totals (Figure 5). The initial feature to be noted is that the papers include more targets than patents (i.e. opposite to the distribution between compounds in Figure 1). We propose three main factors to explain this. The first is that, as a business choice for prioritising SAR extraction, patents are currently limited to the “big-ten” target classes. The second is that papers typically include a broader set of cross-screening results than patents, some of which may have been accumulated by a project team in the years between the drafting of the patents and the papers. The third reason is that papers, certainly from the academic sector, encompass a wide range of bioactive chemistry research (e.g. chemical biology) not directly related to drug discovery. A notable feature of the output from papers is the steady growth rate with a suggestion of post-genomic acceleration after 2001 (Figure 5).

The incremental distribution (Figure 5) follows the same general shape as the cumulative one (Figure 4) but also shows differences. Chief of these are the absolute numbers. Taking the 2010 time point as an example, this means that by 2010, ∼2,500 targets had been published in patents but, during that same year, ∼1,000 proteins had new data published. If the line was to be extrapolated beyond 2010 it would converge towards the 2012 figure in row 12 from Table 1. The incremental plots also look lumpy where the smaller per-year numbers show stochastic deviations. This is because they are derived from only 100s of documents published in any year, compared to the cumulative plot where 1000s of documents smooth the protein growth curve. In the context of shape, it is important to appreciate the difference between the plots. As a hypothetical example, if the global compounds-to-targets fell in 2011 but 20% of the targets published in that year were novel, the accumulated 2011 protein total would show a rise. Note also that Table 1 extends the cumulative target numbers up to the end of 2012 and non-redundantly merges IDs between the two sources. These give 1,987 human proteins from journals and patents, with an equivalent figure from all species as 4,628.

### Single institution analysis for compounds and targets

**Figure 6:**
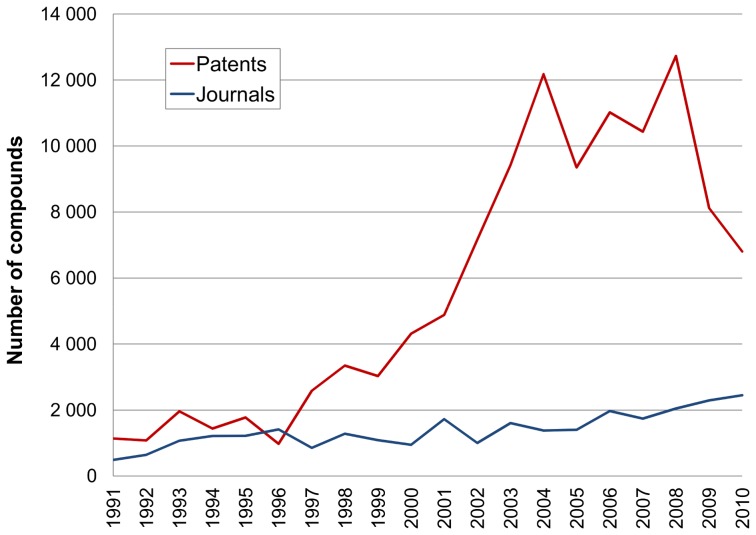
Compounds published by GlaxoSmithKline. Counts by year in patents (red) and journals (blue).

**Figure 7:**
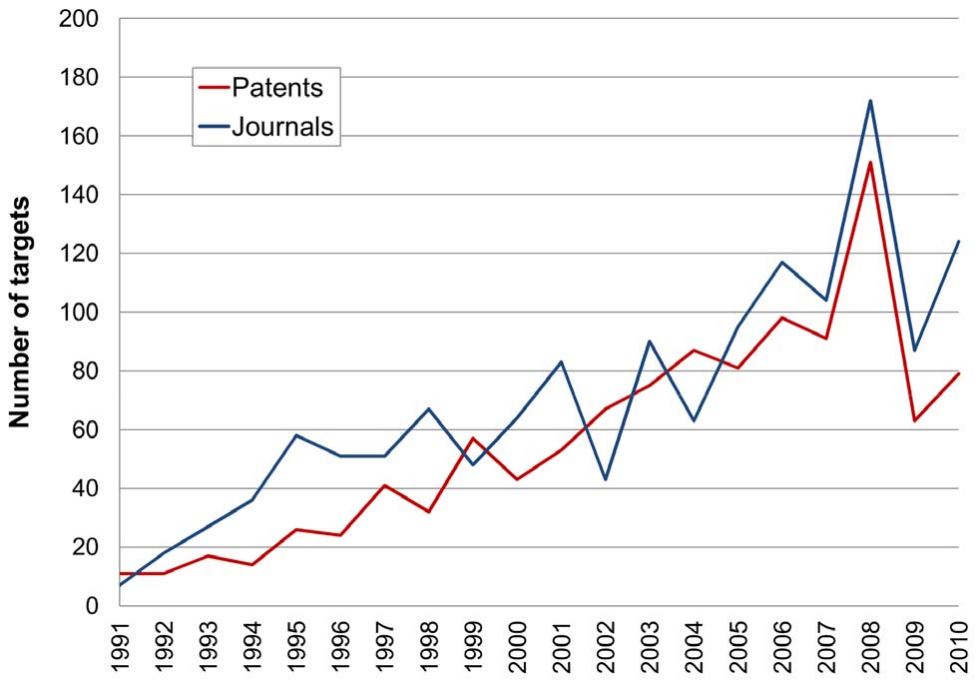
GlaxoSmithKline human targets counts. As extracted from patents (red) and journals (blue) published by year.

As a complement to interrogating the entire data set we can also follow institutional entities across time to reveal details that can be submerged in aggregated data. We chose GSK because, as informative precedent, they have declared the internal statistics of their post-merger target portfolio consolidation exercise [32]. This gave a figure of 328 unique proteins and the surprisingly low total of 37 late-stage active projects in common between GlaxoWelcome and SmithKline Beecham after merging in 2000. The inferences we can make from this published external target data are obviously predicated on internal capacity and priorities. Analogous figures from other companies are rare but the Pfizer annual report from 2006 declared 400 internal projects. However, this dropped to 350 by the following year and, by 2010, was quoted in a different form as 374 projects in both research and development [33]. The plots of targets *vs.* time and compounds *vs.* time for GlaxoSmithKline, including the pre-merger entities, are shown (Figures 6 and 7).

The data in Figure 7 indicates a lower count for GSK ∼100 targets for 2010 but showing a steady rise over two decades. However, this increase may be more apparent than real if the scope of cross-screening has broadened. This appears to be the case for the spike in 2008. Inspection indicates this arises from a small number of patents and papers that included panel screening data against a large number of kinases. On the other hand, external publication would under-count newer target research projects that have not yet generating patent filings. The compound output (Figure 6) tracks the target number but, in parallel with the output from all institutions (Figure 1) appears to flatten out after 2004.

### Target Ranking

From cumulative data we have produced a ranking of proteins *vs.* compounds. As expected, intensively pursued primary drug targets appear towards the top of the list (e.g. F10 was top-ranked with 42,869 compounds) [29]. In this work we extend the approach by resolving this ranking across time (Table 2).

**Table 2:** Ranking of the top-ten targets by year.

rank	1991	1992	1993	1994	1995	1996	1997	1998	1999	2000
**1**	REN	REN	REN	REN	TACR1	**F2**	**F2**	**F2**	**F2**	**F10**
**2**	ALOX5	TBXA2R	ELA2	ELA2	PTGS2	EGFR	TACR1	**F10**	**F10**	**F2**
**3**	ELA2	ALOX5	SRD5A1	ALOX5	PTGS1	TACR1	**F10**	TACR1	MMP1	MMP1
**4**	TBXA2R	ELA2	PTAFR	PTAFR	LTB4R	**F10**	ELA2	MMP1	TACR1	MMP13
**5**	PTAFR	LTB4R	LTB4R	**F2**	ALOX5	TACR2	EGFR	MAPK14	EGFR	KDR
**6**	AKR1A1	C5AR1	ALOX5	TACR1	EGFR	EDNRA	DRD4	MMP13	MMP9	MAPK14
**7**	EGFR	ELA1	SRD5A2	SRD5A1	EDNRA	REN	TACR2	EGFR	ELA2	EGFR
**8**	LTB4R	EGFR	ELA1	EGFR	ELA2	EDNRB	EDNRB	MET	TACR2	MMP9
**9**	CA2	CTSG	TACR1	TACR2	SRD5A1	F7	EDNRA	TACR2	MMP13	TACR1
**10**	SRD5A1	PTAFR	**F2**	ADRA1A	REN	DRD4	MMP1	MMP9	MC1R	MMP2
*cont.*										
**rank**	**2001**	**2002**	**2003**	**2004**	**2005**	**2006**	**2007**	**2008**	**2009**	**2010**
**1**	**F10**	**F10**	**F10**	KDR	KDR	**CNR1**	**CNR1**	**CNR1**	**CNR1**	**CNR2**
**2**	KDR	**F2**	EGFR	**F10**	**F10**	DRD3	HRH3	**CNR2**	HSD11B1	**CNR1**
**3**	**F2**	MAPK14	KDR	**CNR1**	MAPK14	DRD2	**CNR2**	REN	**CNR2**	EDG1
**4**	MMP1	KDR	**F2**	SRC	**CNR1**	KDR	DRD2	BACE1	HRH3	JAK2
**5**	TEK	MMP13	MAPK14	MMP13	PPARA	**CNR2**	DRD3	CTSD	JAK2	KCNH2
**6**	MMP13	MMP1	ADORA2A	TRPV1	**F2**	**F10**	KDR	DRD2	JAK3	JAK3
**7**	CCR3	DRD3	CCR3	MAPK14	TRPV1	REN	OPRM1	HRH3	OPRM1	HTR6
**8**	NPY5R	CRHR1	MMP13	PPARA	PPARG	HTR2C	PPARA	BDKRB1	PIK3CG	PDE10A
**9**	MMP9	NPY5R	CRHR1	MC4R	**CNR2**	HTR2A	HTR6	OPRM1	GCK	PDE5A
**10**	MMP2	CTSS	PPARA	HTR2A	MMP13	BACE1	MC4R	HTR6	JAK1	HSD11B1

Human protein identifiers, as HNC approved symbols ranked in order of the number of compounds linked to them in patents and papers over 20 years. For the targets marked in bold a more detailed longitudinal analysis is presented in a later section.

Inspection of Table 2 reveals a “yo-yo” effect as the relative ranking changes by year. For example, the pole position swaps between renin (1991–1994), then thrombin (1996–1999) followed by Factor X (2000–2003) but all three had dropped outside the top-ten by 2009. Analogously, CNR1 first makes it in to the top-ten for 2004, climbs to the pole position by 2008, along with the paralogue CNR2 in second place. It should be noted that many of the proteins appearing at the top of these rankings have not yet become the targets of approved drugs.

### Tracking Individual Targets

**Figure 8:**
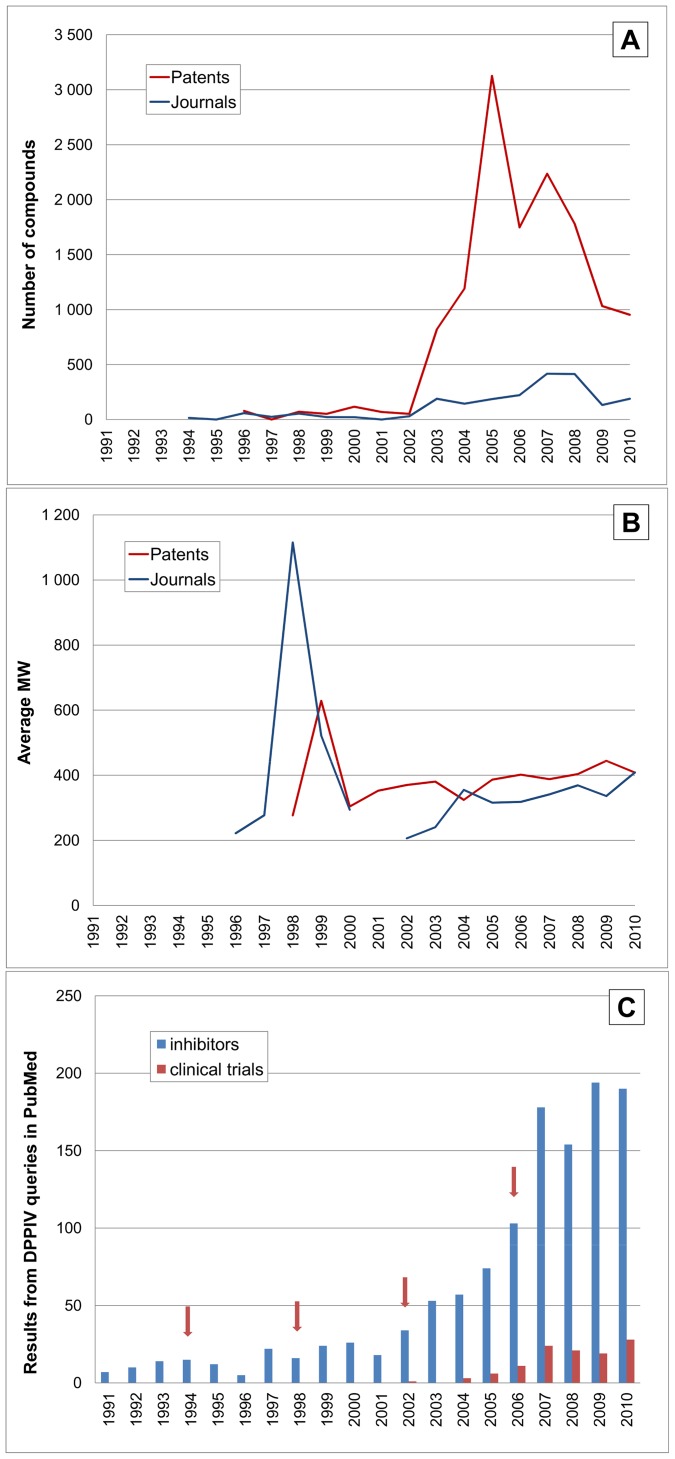
Time courses for DPPIV (UniProt P27487). (**A**) Shows the number of compounds (vertical axis) linked to individual proteins from patents (red) and journal articles (blue). (**B**) Is the average MW for compounds (vertical axis) each year from patents (red) and journal articles (blue). Missing points are years where no documents were extracted. (**C**) Shows queries in PubMed. Abstracts returned by “dpp iv inhibitors” are plotted by year (blue bars). The red bars are clinical trial reports. The arrowed time points are as follows: 1994 early dipeptide inhibitors [52], 1998 the first review related to diabetes therapy [53], 2002 the first clinical trial results [54], and in 2006 the approval of sitagliptin.

A more detailed analysis of the published activity against individual targets can be done by comparing the associated compound outputs by year. We selected six proteases that either, have had drugs against them approved in the last few years, or have candidates in clinical development, together with one non-target protease as a control. Proteases were selected because of the possibility to track MW changes that may be associated with inhibitor progression for this enzyme class. Four of them are colour-coded in Table 2, where shifts in their relative rankings can be seen. We extended this idea to plot the numbers of compounds for patents and papers. This is analogous to the analysis of total outputs (Fig. 1), but for a single target. We sought to corroborate possible interpretations via specific PubMed queries. For example, the query “dpp iv inhibitors” gave 1,583 abstracts. Adding “diabetes” drops this to 1,012 and restricting to the category “clinical trials” drops this further to 302. While “dpp iv inhibitors” returns some false-positives it is possible to pick up papers describing inhibitors as early as 1994. By 1998 their potential use in diabetes was declared in a review article. Consequently, from 1999 to 2003 the PubMed count of “dppiv + inhibitors + diabetes” climbed to 5,7,12 and 39, respectively. For interpretation of timelines from PubMed (or other documents) a “frame shifting” effect needs to be considered. This is because, in competitive intelligence terms, both patent and journal publications are after the fact by several years or more. It should also be noted that these basic literature queries are not completely specific (e.g. not all clinical trials mention inhibitor and reviews of clinical trials are counted along with individual reports). Nonetheless, as we show below, they can generate an interpretable profile. We are thus in a position to compare the three parallel time course for compounds, MW and literature, as shown for DPPIV as the first example (Figure 8).

The compound pattern for DPPIV (Figure 8A) is dominated by a surge from 2003. This continues for patents and papers, approximately in parallel, until a decline in 2007–2008. Note also there is a baseline of published compounds for over a decade before 2003. An explanation is that enzyme inhibitors were being investigated as mechanistic probes before the target hypothesis was established, or at least became “loud and clear” in the1998 review. While the first completed clinical trial report was published 2002 there were earlier disclosures of DPPIV inhibitors entering development. It seems likely that this competitive awareness of target progression and concomitant de-risking induced the surge of follow-on activities that had climbed to 3000 compounds (i.e. at least ∼50 patents) in 2005. The intensity of output around this successful target (reflected in the area under the curve of Figure 8A) is related not only to the approval of no less than four glyptins but also with others in clinical trials. The “frame shifting” effect discussed above implies the first patent filings for these lie within the peak outputs from 2003 to 2009. Because DPPIV has an exopeptidase funnel-shaped substrate pocket rather that an extended endopeptidase binding cleft, the MW of the published inhibitors is close to that of the early generation of cyclic dipeptides (with the exception of a set of large substrate derivatives published in 1998). Thus the average falls to ∼200 but then rises to ∼400 (Figure 8B) and is close to the 407 for sitagliptin. Note that by the time sitagliptin had been approved by the FDA as first-in-class in 2006, the total numbers of compounds were already declining.

**Figure 9:**
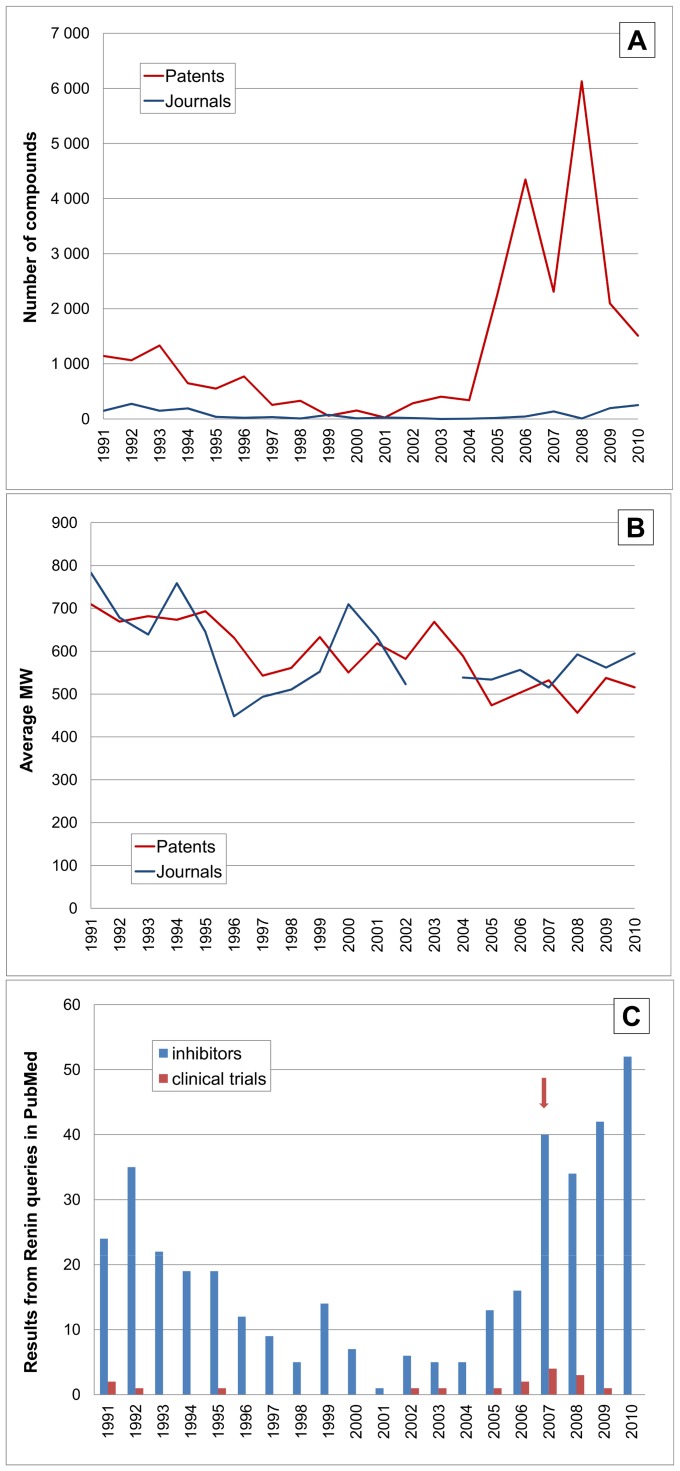
Time courses for renin (REN, UniProt P00797). The horizontal axes and line labels are the same for Figure 8 except for the vertical scales. (**A**) Shows the number of compounds linked to renin. (**B**) Shows the average MW for compounds. (**C**) shows queries in PubMed (in this case “renin inhibitors” needed to be constrained as a phrase query to preclude “renin-angiotensin system” false positives). The arrowed time point is the 2007 FDA approval of aliskiren.

The analogous set of results for renin (Figure 9) show different profiles to DPPIV and thus require alternative explanations. The most notable features are that the journal publications are distinctly biphasic and, to an extent, this is also mirrored by the compound output with the same minimum around the year 2000.

A clue to the observed biphasic pattern lies in the key statement from a 2001 review “all renin inhibitor development programs have been closed” [34]. Some of these closures were related to the clinical failure reports in the early 1990s preceded by the 1991–1993 compound output phase (Figure 9A). But from this activity trough, the pick-up in clinical trial reports may be related to the progression of aliskiren (Figure 9C). Here again, the early intelligence effect probably resulted in the 2005–2009 follow-on patenting surge (similar in magnitude to that for DPPIV in Figure 9A) but the corresponding publication increase is displaced by approximately one year. For this target we do see a distinct fall in average MW by ∼200 (Figure 9C). As summarised in the Wikipedia entry [35], renin inhibitors have passed through three distinct inhibitor design generations of I) peptide analogues, II) peptide mimetics, and III) non-peptidic small molecules. Thus, our post-1991 results are predominantly capturing the third generation within which aliskiren has a MW of 552.

**Figure 10:**
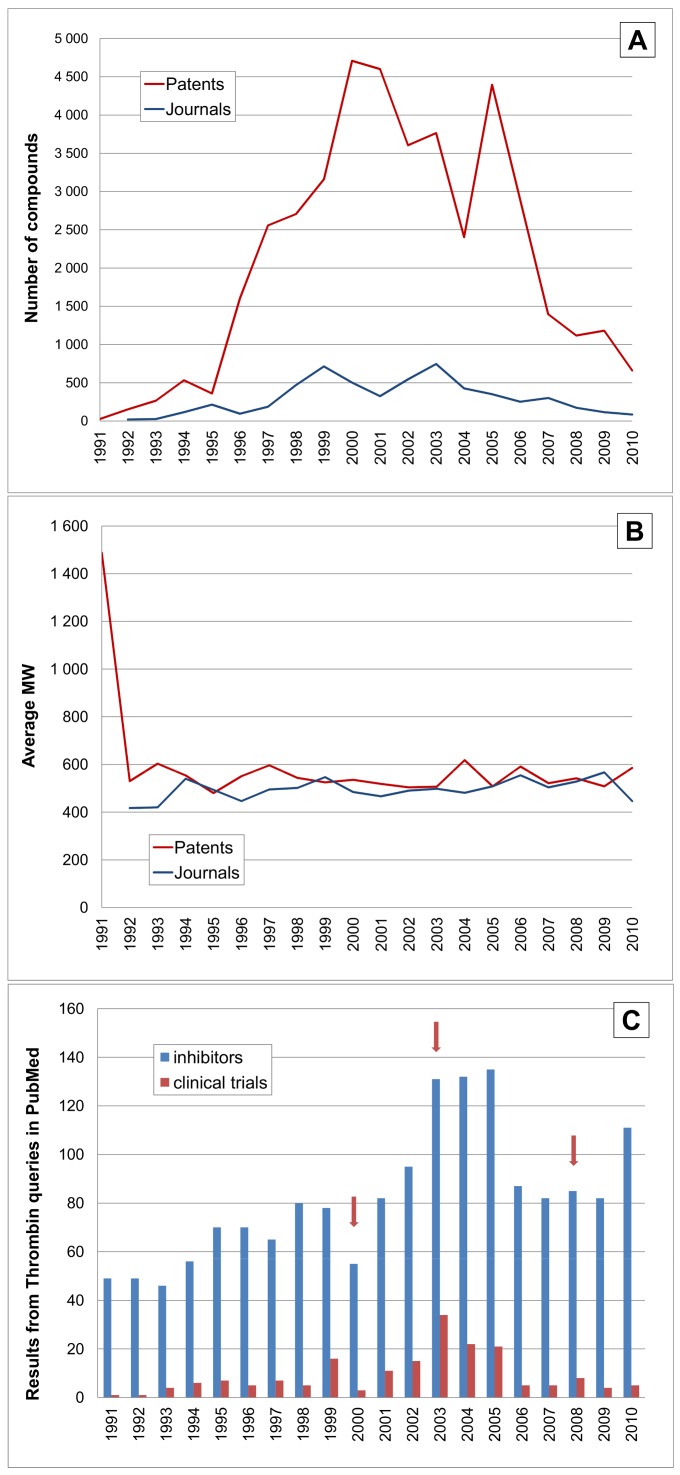
Time course for thrombin (F2, UniProt P00734). The horizontal axes and line labels are the same for Figure 8 except for the vertical scales. (**A**) Shows compounds linked to thrombin. (**B**) shows average MW for compounds. (**C**) Shows queries in PubMed for inhibitors and clinical trials (but the pre-2003 and some subsequent results are associated with hirudin analogues and other non-small molecule inhibitors). The arrowed timelines are the approval of atroban as an injectable in 2000, the first clinical trial of an oral inhibitor, ximelagatran, in 2003 and the FDA approval of dabigatran in 2008.

The next two targets are coagulation serine proteases with a long history of biological inhibitor investigations but the development of small-molecule direct inhibitors as clinical candidates is more recent. The time courses for thrombin are shown in Figure 10. In contrast to the sharper peaks for DPPIV or renin, thrombin shows a sustained output for over 15 years. The displacement of the three peaks for journals by approximately two years with respect to the patent output peaks, suggests this could be a patent-then-publish effect. This protease also shows a higher proportion of structures from papers although this is still well below the patent output. As noted in the legend to Figure 10C the publication profile cannot be made specific for small-molecule only inhibitors without manual curation, but there does seem to be a peak after the initial first-in-class clinical phase I progression of ximelagatran (CID 9574101) in 2003 [36]. By the time dabigatran (CID 9578572) was approved in 2008 [37], the numbers of patented inhibitors had noticeably declined (Figure 10A). Note also that, subsequent to the publication of some 1991 patents on large peptide inhibitors, the average MW stays close to ∼500 with the clinical pro-drugs ximelagatran and dabigatran coming in at 474 and 472 respectively.

**Figure 11:**
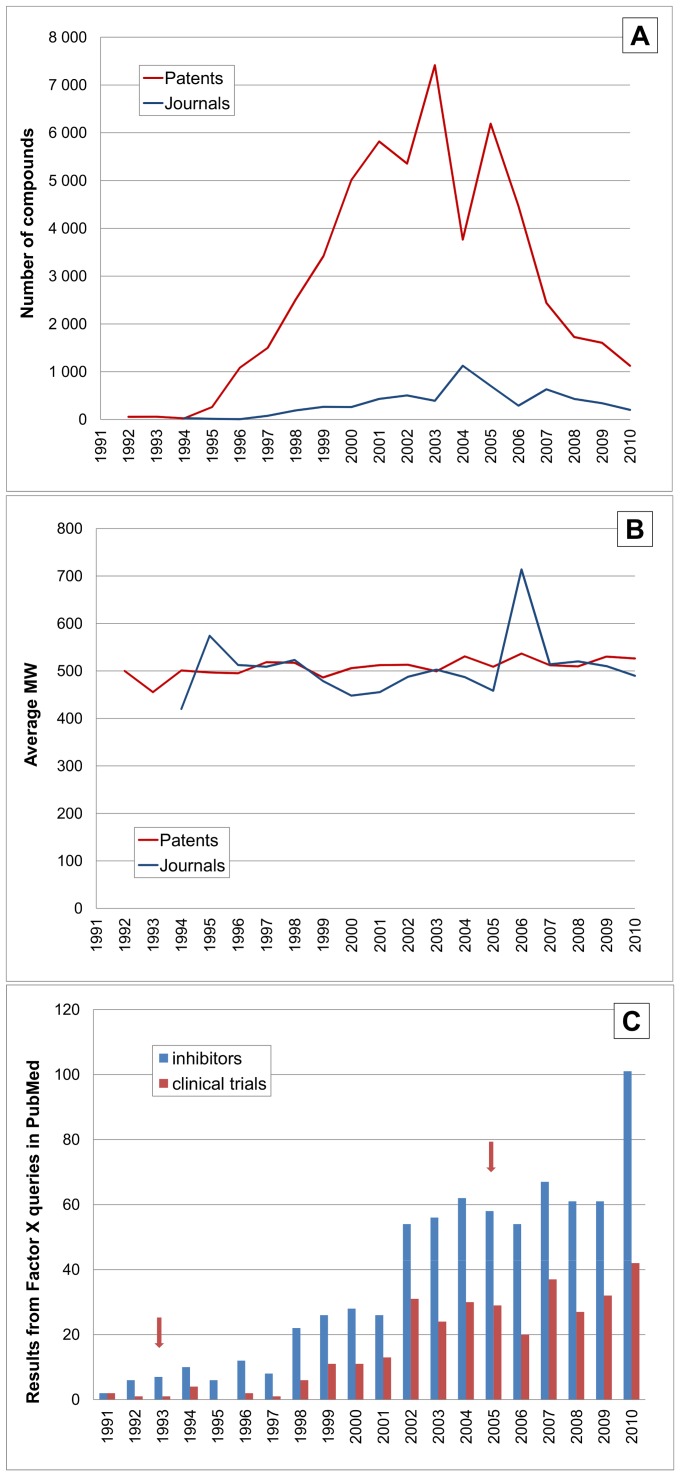
Time courses for Factor X (F10, UniProt P00742). The horizontal axes and line labels are the same as for Figure 8 except for the vertical scales. (**A**) Shows compounds linked to F10. (**B**) shows average MW for compounds. (**C**) Shows queries in PubMed for inhibitors and clinical trials the results are associated with heparin analogues and other non-small molecule inhibitors. The arrowed timelines are for the first PDB structure in 1993 and first clinical trials of direct inhibitors in 2005.

The next target, Factor X (strictly Xa, Figure 11) shows a similar extended profile to thrombin but covers more compounds. The large compound numbers are reflected in the high ranking of both targets (Table 2) which can be attributed to both their chemical tractability and “popularity”. However, compared to thrombin. F10 shows the highest peak output concentrated between 2000 and 2006. This precedes the first clinical trials for rivaroxaban in 2005 but this was approved for certain indications for Europe in 2008 before the FDA in 2011 (CID 9875401) [38]. Here also, the literature profile specificity is confounded by early work on biological inhibitors, such as tick anticoagulant peptide (rTAP) and recombinant antistasin (rATS) heparinoids, as well as clinical trials for injectable agents. Nonetheless, the extraction of compounds from 1994 onwards (and inspection of selected titles) indicates the majority of the later papers are describing orally administered direct Xa inhibitors. The extended rise in patent compounds could be related to the first PDB structure in 2003 and consequent structure-aided design including inhibitor co-crystallisation.

There is an overall similarity of pattern between both these coagulation protease targets that could have a causative basis. Beyond postulating this to be simply coincidental (i.e. via many organisations working on them over similar time periods) there is another explanation that has already been pointed out from consideration of their cumulative ranking and compounds-in-common [29]. This can be termed parologous coupling where lead compounds directed against either as primary target are cross-screened for specificity against homologous human enzymes as a matter of course (some compounds may even have been pursued as dual inhibitors but not a high proportion). This effect may have also caused the average MW to converge at ∼500 (Figures 10B and 11B) but rivaroxaban comes in below this with a MW of 436.

**Figure 12:**
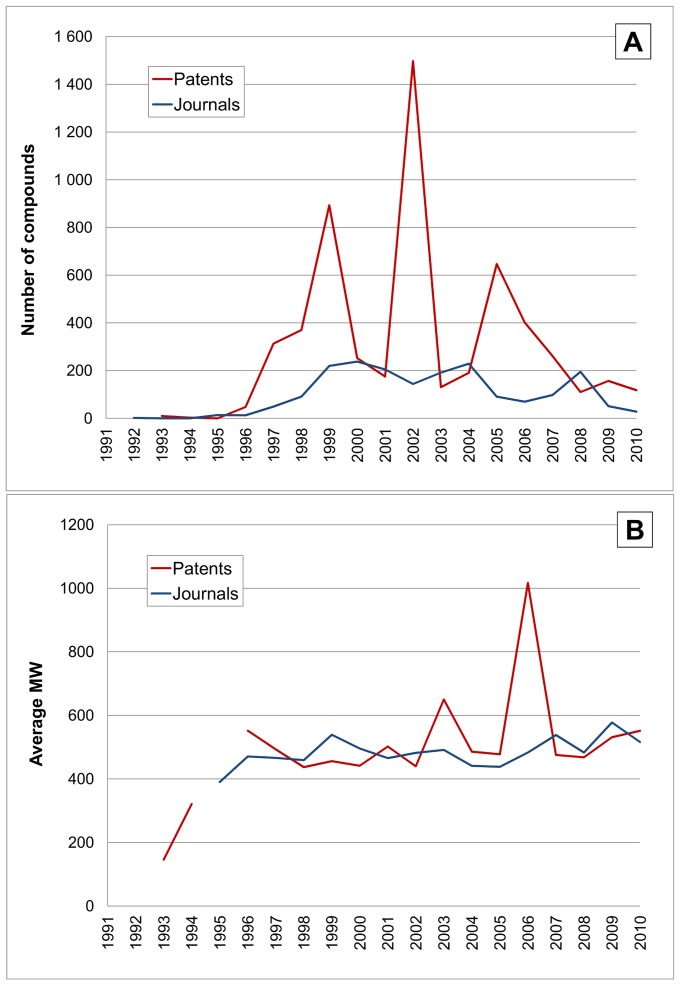
Time courses for Trypsin (PRSS1, UniProt P07477). The horizontal axes and line labels are the same for Figure 8 except for the vertical scales. (**A**) Shows compounds linked to Trypsin. (**B**) shows average MW for compounds.

We examined the parologous coupling effect further by extracting data for trypsin (PRSS1, EGID 6544). While it was not possible to specifically identify small-molecule inhibitors via PubMed, the output profile is shown below in Figure 12. While trypsin is a drug target for pancreatitis, no recent major efforts in development of specific low-MW inhibitors have been reported. However, it remains a significant “target” in the compound-to-protein mapping sense because, as classical serine protease mechanistic exemplar, it is widely used in specificity cross-screening not only for thrombin and F10 but also other coagulation protease targets. The three significant output peaks in Figure 12 would seem to support this. There is also an implication of parallel tracking, for example the 2002 peak is close to the maximum output associated with F10 (Figure 11A).

**Figure 13:**
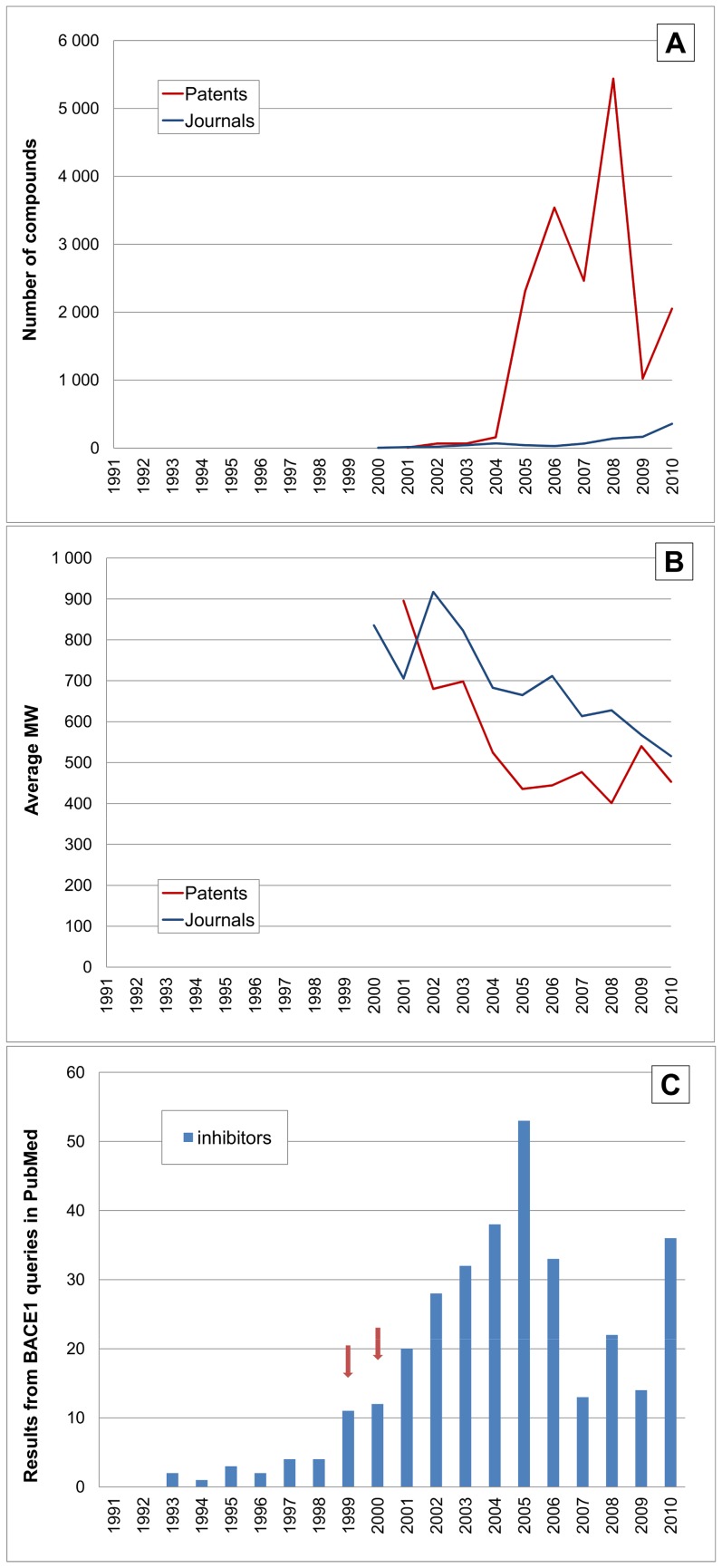
Time courses for BACE1 (UniProt P56817). The horizontal axes and line labels are the same for Figure 8 except for the vertical scales. (A) Shows compounds linked to BACE1. Note here the profile for papers looks low because it was plotted on the same scale as used for patents. (**B**) shows average MW for compounds. (**C**) Shows queries in PubMed for BACE1 inhibitors; those before 1999 are spurious because, at default settings, gene name queries record back-mappings, in this case investigations on inhibiting beta-secretase activity where the MeSH system added the BACE1 gene term retrospectively. The arrowed timelines are for the identification of BACE1 in 1999 and first PDB structure in 2000.

The profiles for the second aspartyl protease in this set, BACE1, look very different to the targets described so far (Figure 13). Much of the difference is due to BACE1 being a younger target since the protein was only identified in 1999 (although a consensus on the target concept for DPPIV in diabetes only emerged a couple of years before this) [39]. This “starting gun” effect for effectively synchronous target validation by independent groups, followed quickly by the first PDB structure by 2000, could be causatively related to the rapid rise in compound output starting in 2004. Uniquely, we see a distinct drop in average MW from the peptide analogues published in the early papers down to inhibitor leads in the 400–500 range by the time patent publications had taken off in 2005 (i.e. the peptidomimetic-to-small-molecule shift). The only clinical candidates with declared structures and reported phase I results are LY2811376 (but progression was halted) and AZD3839 from 2011 and 2012 respectively (CID 60210951, CID 44251605) [40], [41]. These come below the patented lead average MW at 433 and 320, respectively.

**Figure 14:**
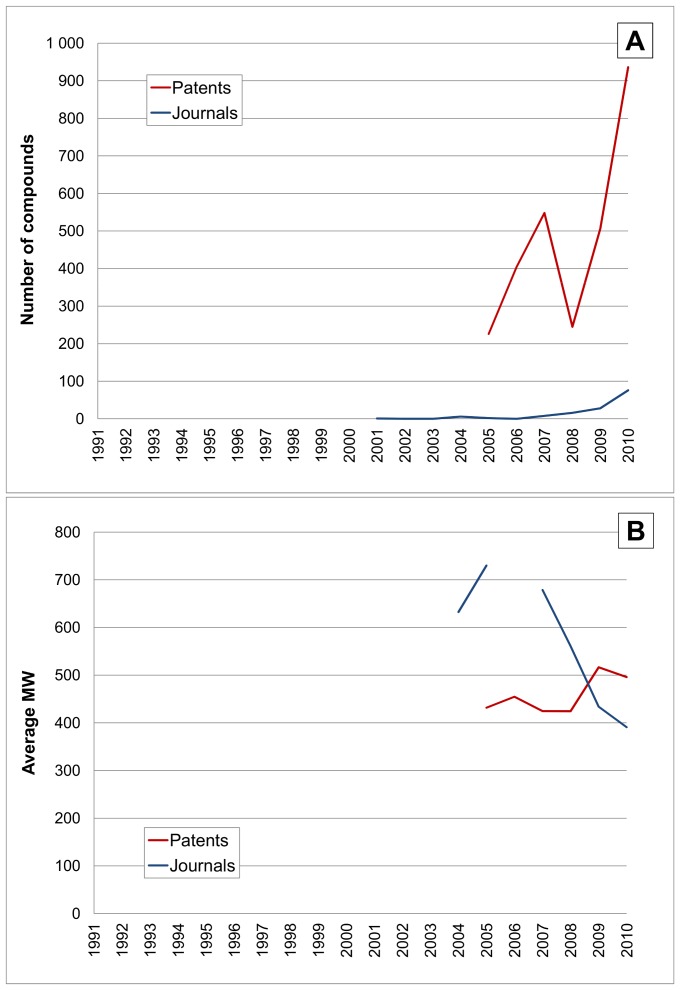
Time courses for BACE2 (UniProt Q9Y5Z0). The horizontal axes and line labels are the same for Figure 8 except for the vertical scales. (**A**) Shows compounds linked to BACE2. (**B**) shows average MW for compounds. There are no specific inhibitor publications retrieved with PubMed queries.

We chose to add BACE2 to the target examples because it exemplifies a special case of the parologous relationship (Figure 14). The pattern runs approximately parallel to BACE1 but shows a strong rise in 2010. Analogous to the situation with thrombin and F10 the BACE2 compound profile would be expected to represent specificity cross-screening but in this case BACE2 had no prior history of being a drug target and therefore has no inhibitor matches in PubMed. However, this situation changed abruptly in 2011 when BACE2 was declared as potential drug target for diabetes in a paper with Roche co-authors [42]. Unsurprisingly, Roche patents specifying BACE2 inhibitors for diabetes first appeared in 2010 and have produced the strong upward spike as this enzyme transitions from cross-screening to a primary target (Figure 13A) [43].

## Discussion

As far as we can determine this is the first time a large global decrease has been reported in terms of target-associated chemistry from patents. It thus deserves interpretive comment even if the correlations we might consider cannot establish causality. However, reports of declining research output have indirect precedents (i.e. not derived from compound counts *per se*). The first is the description of an enterprise chemical data integration system that included a chart in which WIPO medicinal chemistry patents showed a plateau from 2004 to 2010 [26]. The second was a detailed analysis of chemical properties in patents from 18 pharmaceutical companies that reported a decline in targets from 2006 to 2010 compared to the preceding five years [27]. The trend of the slower and more linear increase for journal compound output in Figure 1 parallels that recently reported for *Journal of Medicinal Chemistry* that includes a subset of the same data used here from MCD [28]. This was recorded over a longer time span but is more discontinuous being derived from a single journal.

Our patent office data corroborates the fall in SAR-linked output. Because we have recorded a concomitant rise on a per-document basis this infers the number of patents has fallen faster than compounds. The equivalent output from papers has not declined, suggesting that drug discovery operations have continued to publish structures, including those derived from earlier patents. Queries in PubMed that included both an affiliation from a large pharmaceutical company and a major medicinal chemistry journals (results not shown) suggested a slowdown for papers in the last five years but not as pronounced as that for patents in Figure 2. Because the ratio of structures in patents to papers is ∼4:1 we might expect that medicinal chemistry journal output is being sustained in the face of a patenting decrease. While there is an obvious parallel between the declines in research compounds and new drug approvals it should be noted that the two outputs are separated by up to 10 years. In addition, NMEs per-year began their downward inflection approximately in 1997–1998 whereas our results show that research output began to fall between 2005 and 2006. The implied contraction in medicinal chemistry productivity is counter-intuitive in two respects. The first is that it coincides with technical advances that would have been predicted to sustain or even exceed the acceleration seen between 2000 and 2005 [44]. The second is that a bibliometric analysis of papers, genes and diseases suggests that the academic research activity, upon which innovations in drug discovery are largely predicated, continues to show an upward trend [45].

While consideration of all possible causative factors is outside the scope of this work it could be speculated that the dominant causal effect on global output is mergers and acquisition activity (M&A) among pharmaceutical companies. The consequences of this include target portfolio consolidations and the combining of screening collections. This also reduces the number of large units competing in the production of medicinal chemistry IP. A second related factor is less scientists engaged in generating output. Support for the former is provided by the deduction that NME output is directly related to the number of companies [1] and for the latter, a report that US pharmaceutical companies are estimated to have lost 300,000 jobs since 2000 [46]. There are other plausible contributory factors where finding corroborative data is difficult but nonetheless deserve comment. Firstly, patent filing and maintenance costs will have risen at approximately the same rate as compound numbers. Therefore part of the decrease could simply be due to companies, quasi-synchronously, reducing their applications to control costs. While this happened for novel sequence filings over the period of 1995–2000, we are neither aware any of data source against which this hypothesis could be explicitly tested for chemical patenting nor any reports that might support it. Similarly, it is difficult to test the hypothesis of resource switching from “R” to “D” as a response to declining NCE approvals. Our data certainly infer the shrinking of “R” but there are no obvious metrics delineating a concomitant expansion of “D”. A third possible factor, a shift in the small-molecule:biologicals ratio in favour of the latter is supported by declared development portfolio changes in recent years but, here again, proving a causative coupling is difficult.

Despite the “glass-half-empty” implications, the decline in compound output *per se* need not be interpreted entirely in negative terms. Specifically, our analysis implies nothing about the overall quality of this still very large pool of lead-like compounds from which the drug candidates of the future have been, and are being, selected. For example, some of the decline could be attributed to the “better than the Beatles” problem where the historically success of older drugs means that research efforts are more innovative in being re-directed away from the easy targets towards a smaller pool of newer and less chemically tractable ones [5]. Another factor that could be filtering down the numbers is the steady improvement of compound quality through advances in *in vitro* and *in silico* physicochemical profiling [47]. It should also be noted that the global productivity peak we observed for 2004–2005 should now be feeding through to clinical development and could thus result in an increase in NCEs over the next few years. While it may be too early to tell, there has been a notable rise in 2011 FDA approvals that appears to be sustained for 2012 [48], [49].

In contrast to compounds the sustained increase we observe for targets presents a more optimistic picture. This indicates that over 3500 proteins from patents and papers now have chemical modulation data and ∼100 new ones are added each year. In addition, SAR results for over 800 have appeared in patents, each year, from 2006 to 2010. If a restriction to human-only proteins had been applied the numbers would be approximately halved but the distributions are essentially the same (data not shown). While this shows the drug discovery community found new proteins to test small-molecules against at a steady rate over the last 20 years, the recent flattening out infers the collective capacity to may be reaching a maximum, possibly limited by the same factors described for compounds. However, as previously pointed out, there are two principal confounding factors [29]. The first is that the majority of effort is focused on a small number of targets (e.g. those in Table 2). The second is that a general increase in paralogue and orthologue cross-screening for specificity testing produces an apparent increase in compound-to-protein data mappings, whereas the number of primary targets being pursued (i.e. roughly equivalent to project counts in pharmaceutical companies) is much lower.

The analysis for GSK cannot be taken as average representation of all individual large R&D organisations but nonetheless provides an informative example. The results exhibit stochastic effects because extractions from smaller numbers of documents can fluctuate significantly on a yearly basis rather than showing a smooth aggregate output. Nevertheless, the discernible trends seem to mirror those we observe overall. Due to factors such as proteins in common between therapeutic areas, project counts *per se* may be higher than primary targets. There are other confounding effects such as where protein complexes are the experimental targets or projects based on black-box phenotypic screening. Nevertheless, such figures as we present, not only suggest the larger pharmaceutical companies were actively screening between 200 and 300 proteins but also that this has shrunk over the last five years.

Tracking individual proteins across time provides a level of resolution for target-specific trends that, as far as we can establish, is unique. Three aspects of our results surprised us. The first was the intensity of “follow on” signals where global outputs jumped by two-fold or more within a year. The second was the pronounced differences in patterns, for example the biphasic one for renin, a long broad spread for thrombin and the late but strong peak for BACE1. While it is difficult to prove causality for correlations some likely factors are part and parcel of competitive intelligence. They are thus openly acknowledged as signals for progression and de-risking of particular target/disease combinations. We have indicated some that are discernible in the literature, such as the first protein-ligand structures, major validation reviews, successful Phase 1 studies and first-in-class approvals. A third aspect was also striking. This was that all four target examples (with the obvious exception of BACE2) had all significantly declined in terms of compound numbers by 2010. As a specific example, while it would be an exaggeration to say the community had “given up” on Factor X inhibitor research, patenting has clearly fallen precipitously. The inference here is that, by the time the global development pipeline output for a popular target becomes crowded with clinical candidates, there remains little commercial justification for further research efforts to produce an *n*-th in class drug.

The generation of new detailed insights into the processes of drug discovery has inherent utility for guiding its practitioners. There are clearly options to extend our interrogation, for example profiling an extended range of molecular properties (e.g. scaffolds) for individual targets against time, as well as selected target groupings (e.g. proteases by mechanistic class). However, by definition, such analyses are retrospective, so the question arises as to how this can be exploited predictively. In our opinion there is valuable scope for rational extrapolation based on the types of results we have described. This is certainly the case for hypothesis testing in what could be termed molecular competitive intelligence. For example, one of the most pressing needs is in the area of target validation. In this regard we have observed that a steady collective decline in compounds for a target can be an indicator of success (i.e. declining commercial opportunity), intractability or failure (i.e. a consensus de-validation). While it is not always obvious which of these outcomes is most likely, there are cases where a comparative retrospective analysis may provide clues. For example, early compound-chemotype-target profiles from early patents could be connected and tracked through to Phase II and III outcomes. Further analysis and prediction of how drug discovery operates based on the data it actually generates will be important as this global undertaking adapts to the challenges and opportunities of the future.

## Methods

The databases used have been described in detail elsewhere ([29] and references therein) but a short outline is as follows. The GVKBIO Medicinal Chemistry Database (MCD) is populated via large-scale expert extraction of structure-activity relationships (SAR) from a set of 120 medicinal chemistry journals. These predominantly report results of drug discovery research and are selected on the basis of a high per-issue yield of extractable SAR. The Target Class Databases (TCD) are populated analogously to except that the relationships are extracted from patents covering the target classes of kinases, GPCRs, proteases, nuclear hormone receptors, ion-channels, transporters, lipases, phosphatases, oxidoreductases and transferases. Because of their numerical dominance in our compound-to-protein results it is important to understand the SAR extraction capping rules. The consistent application of these across the large patent corpus is essential for the consistency of our results. Exemplified structures linked to targets via quantitative assays, typically IC50 or Ki values, have no extraction limit. If the linked data is ranged (e.g. binned between 0.01 and 0.1 uM) extraction is capped at 500 examples. In cases where activity is only qualitatively specified (e.g. using a star rating) extraction is limited to 100 structures.

Data in MCD and TCD is predominantly from the larger pharmaceutical companies. However, there is a long tail of hundreds of smaller commercial entities who also publish papers and patent [7]. Academic output is also captured and, for the selected high SAR-content journals, curators make no distinction (other than recording the institutional affiliation as metadata) between papers reporting drug discovery, mechanistic enzymology, chemical biology or other bioactive chemistry domains. Queries for this work were carried out using SQL via the GOSTAR database schema. This integrates MCD and TCD with other databases and is instantiated both at GVKBIO and internally (with some customisation) at AstraZeneca. Compounds were counted via an internal identifier for unique structures. These were split between papers and patents using the appropriate table join in MCD and TCD, respectively. These tables were also used to select year-on-year and cumulative (year X to year Y) time points based on the publication date of the documents from which the structures were extracted. Targets were counted via their distinct Entrez Gene IDs but these could also be restricted by species.
